# A Novel A‐Kinase‐Anchoring Protein 9 Variant in Premature Coronary Artery Disease: A Case Series

**DOI:** 10.1002/mgg3.70159

**Published:** 2026-01-23

**Authors:** Yuemiao Jiao, Minxian Wang, Guifen Qiang, Li Zhao, Ziwei Xi, Yue Yu, Chengqian Yin, Guangyuan Song

**Affiliations:** ^1^ Department of Interventional Center of Valvular Heart Disease Beijing Anzhen Hospital, Capital Medical University Beijing China; ^2^ National Genomics Data Center, China National Center for Bioinformation Beijing China; ^3^ Beijing Institute of Genomics Chinese Academy of Sciences Beijing China; ^4^ State Key Laboratory of Bioactive Substance and Function of Natural Medicines Institute of Materia Medica, Chinese Academy of Medical Sciences and Peking Union Medical College and Beijing Key Laboratory of Drug Target and Screening Research Beijing China; ^5^ School of Sport Science, Beijing Key Laboratory of Sports Function Assessment and Technical Analysis Beijing Sport University Beijing China

**Keywords:** AKAP9, familial premature coronary artery disease, whole‐exome sequencing

## Abstract

**Background:**

Familial premature coronary artery disease (CAD) is often associated with genetic variants. This study investigated potential causal variants in a Chinese pedigree with premature CAD.

**Methods:**

In total, nine family members were included in the study (six CAD patients and three unaffected controls). Whole‐exome sequencing (WES) was performed on six family members (including four patients and two unaffected controls), and the candidate variant was further validated by Sanger sequencing in four individuals.

**Results:**

A strong linkage between c.6406C>G (p.Gln2136Glu; NM_005751.5) in *AKAP9* (A‐KINASE ANCHOR PROTEIN 9; OMIM 604001) and premature CAD was detected in the pedigree. Functional analysis revealed that the c.6406C>G variant in *AKAP9* decreased the interaction between AKAP9 and PRKAR2A. This association was first detected in premature CAD patients.

**Conclusions:**

Our findings indicate that c.6406C>G in the *AKAP9* gene could be a causal variant for premature CAD in the Chinese population.

## Graphical abstract

AKAP9 Q2136E mutation disrupts AKAP9–PRKAR2A interaction and may contribute to early‐onset CAD.

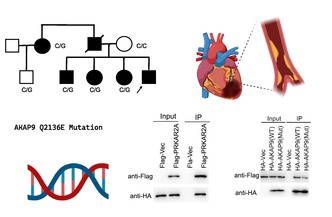

## 1 Introduction

Cardiovascular disease (CVD) remains a leading contributor to the global burden of disease and represents the leading cause of death worldwide (Roth et al. 2020). Premature CAD shows a strong tendency for familial clustering and genetic predisposition (Marenberg et al. 1994; Hakansson et al. 2025). However, there is no universally accepted definition of premature CAD. Reported age thresholds vary across studies, with most using cutoffs of 45 or 55 years, while earlier studies often applied 65 years as the upper limit (Mousavi et al. 2025; Collet et al. 2019). To maintain consistency with the thresholds most frequently cited in recent literature, we defined premature CAD as the onset of cardiovascular events before 45 years in men and before 55 years in women (van Loon et al. 2012). Nevertheless, the current genetic landscape of premature CAD remains far from complete. By investigating a Han Chinese family with multiple cases of premature CAD through whole‐exome sequencing (WES), we aimed to contribute additional evidence to the growing genetic map of this disease.

## 2 Materials and Methods

### 2.1 Ethics Statement

This study was approved by the Ethics Committee of the Beijing Institute of Genomics (approval number: 2023H010). All procedures were performed in accordance with the ethical standards outlined in the Declaration of Helsinki (1975) and its later amendments. Prior to participation, all patients and controls provided informed consent.

### 2.2 Subjects

**FIGURE 1:**
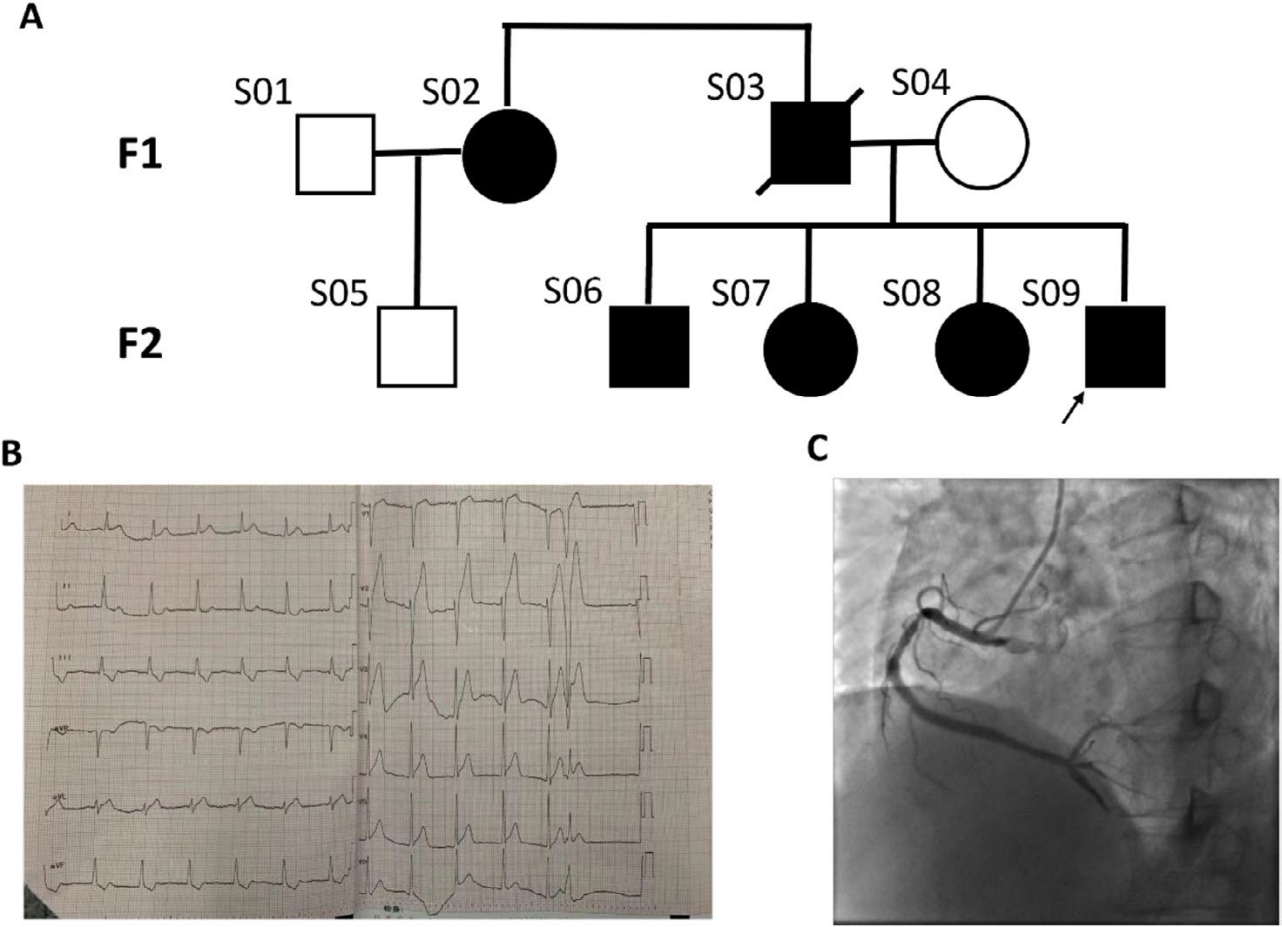
The proband and her pedigree. (A) Family chart of premature coronary heart disease in China. The black arrow indicates the proband. (B) Typical ECG of S09 with premature CHD. (C) This is a typical coronary angiography in S09 with premature coronary heart disease.

A total of nine individuals within two generations of the same Han Chinese family were recruited in this study. Among them, six were diagnosed with CAD including one who had died of acute myocardial infarction and five living patients (Figure 1A). The three family members without cardiovascular history or diagnosis served as internal controls. Of the six affected individuals, four met the standard criteria for premature CAD (cardiovascular events occurring before age 45 in males and before age 55 in females). One additional patient was diagnosed with CAD at the age of 56, but based on the clinical history suggesting earlier onset of symptoms, we considered this individual as having premature CAD, as further discussed in the Discussion section. The clinical history of the deceased patient was obtained from medical records and family reports. Four of the living patients underwent clinical evaluation and blood sampling at Beijing Anzhen Hospital in 2019, while blood samples from the other two living patients were collected by trained laboratory personnel after obtaining written informed consent. The diagnosis and severity of CAD were assessed by an experienced cardiologist on the basis of angiographic findings. The three healthy controls did not show any signs or symptoms of cardiovascular events. All patients and controls included in the study signed informed consent before the start of the study (Table 1).

**TABLE 1:** Clinical history and treatment summary of Chinese premature CVD family members.

Number	Age(years)	Age of onset(years)	Clinical diagnosis	Treatment history
S01	68	—	—	—
S02	69	55	Unstable angina	Interventional therapy 1 time
S03	Died at the age of 51	50	Acute myocardial infarction	Rescue not prompt
S04	80	—	—	—
S05	37	—	—	—
S06	58	56	Acute myocardial infarction	Cardiac bypass surgery, interventional therapy 1 time
S07	54	52	Unstable angina	Interventional therapy 1 time
S08	53	51	Unstable angina	Cardiac bypass surgery
S09	46	44	Acute myocardial infarction	Interventional therapy 4 times

### 2.3 Clinical Parameters and Biochemical Measurements

Baseline characteristics, including smoking and drinking habits, sex, age, height, weight, myocardial infarction (MI), hypertension, and diabetes mellitus, were recorded (Table 2). In this study, we adopted the National Institute on Alcohol Abuse and Alcoholism (NIAAA) definition of low‐risk alcohol use: for men ≤ 65 years of age, no more than 4 standard drinks on any single day and no more than 14 drinks per week; for women and for men ≥ 66 years of age, no more than 3 standard drinks on any single day and no more than 7 drinks per week (1 standard drink≈14 g of ethanol) (Piano et al. 2025). We define smokers as those who have smoked continuously or cumulatively for 6 months or more in their lifetime. Whole blood was collected by vacuum blood collection without anticoagulant and centrifuged at 1500× g for 15 min. Serum concentrations of low‐density lipoprotein cholesterol (LDL‐C), triglycerides (TG), high‐density lipoprotein cholesterol (HDL‐C), total cholesterol (TC), serum creatinine (SCr), fasting blood glucose (FBG), lipoprotein(a) (Lp(a)), and homocysteine (Hcy) were determined in the morning after fasting for at least 8 h.

**TABLE 2:** Clinical characteristics of Chinese premature CVD family members.

Variable	S06	S07	S08	S09	Reference value
Gender	Male	Female	Female	Male	
Onset age of CAD	56	52	52	44	—
Hypertension	No	No	No	Yes, 15 years	—
Diabetes	No	No	No	No	—
Smoking	No	No	No	No	—
Drinking	No	No	No	No	—
FBG (mmol/L)	4.65	5.36	5.59	5.33	3.90–6.10
TG (mg/dL)	49.60	132.86	69.97	90.34	0–150
TC (mg/dL)	138.05	129.16	149.27	150.81	120–200
HDL‐C (mg/dL)	67.67	37.90	53.36	38.28	40–60
LDL‐C (mg/dL)	72.70	75.02	82.75	91.26	50–120
Scr (μmol/L)	68.4	65.7	59.7	53.1	57.00–111.00
Hcy (μmol/L)	15.0	35.7	9.30	21.0	6–16
Apo‐A1 (mmol/L)	1.15	1.17	1.41	1.54	1.05–1.75
Apo‐B (mmol/L)	0.71	0.84	0.81	0.84	0.60–1.40
LP(a) (mmol/L)	0.61	0.90	0.69	1.13	0.00–0.30
sdLDL‐C (mmol/L)	0.44	0.66	0.62	0.75	—
ALT (U/L)	15.4	22	90.2	25.4	9.00–60.00
AST (U/L)	17.6	19.9	22.3	33.1	15.00–45.00
CK (U/L)	111.1	42	70.3	71.9	38.00–174.00

Abbreviations: ALT, alanine aminotransferase; Apo‐A1, apolipoprotein A1; Apo‐B, apolipoprotein B; AST, glutamic–pyruvic transaminase; CK, creatine phosphate kinase; FBG, fasting blood glucose; Hcy, homocysteine; HDL‐C, high‐density lipoprotein cholesterol; LDL‐C, low‐density lipoprotein cholesterol; LP(a), lipoprotein a; SCr, serum creatinine; sdLDL‐C, small, dense LDL cholesterol; TC, total cholesterol; TG, triglyceride.

### 2.4 Whole Exome Sequencing and Bioinformatics Analysis

#### 2.4.1 Sample Selection and Variant Filtering Strategy

WES was performed in six family members, including four individuals with confirmed CAD (S02, S06, S07, S08) and two without a cardiovascular history (S04 and S05). For the initial variant filtering, the four affected individuals were compared with one unaffected older family member (S04) as an internal control to generate a list of candidate variants (Table S1). S05 was not included as a control in this filtering step because of the younger age and the possibility of developing CAD later in life. Variants were prioritized based on segregation with the disease phenotype, rarity in public databases, predicted functional impact, and previous associations with CVDs. Candidate variants were subsequently validated by Sanger sequencing in four available individuals (S04, S06, S07, S08).

#### 2.4.2 WES Experimental Procedures and Data Analysis

**FIGURE 2:**
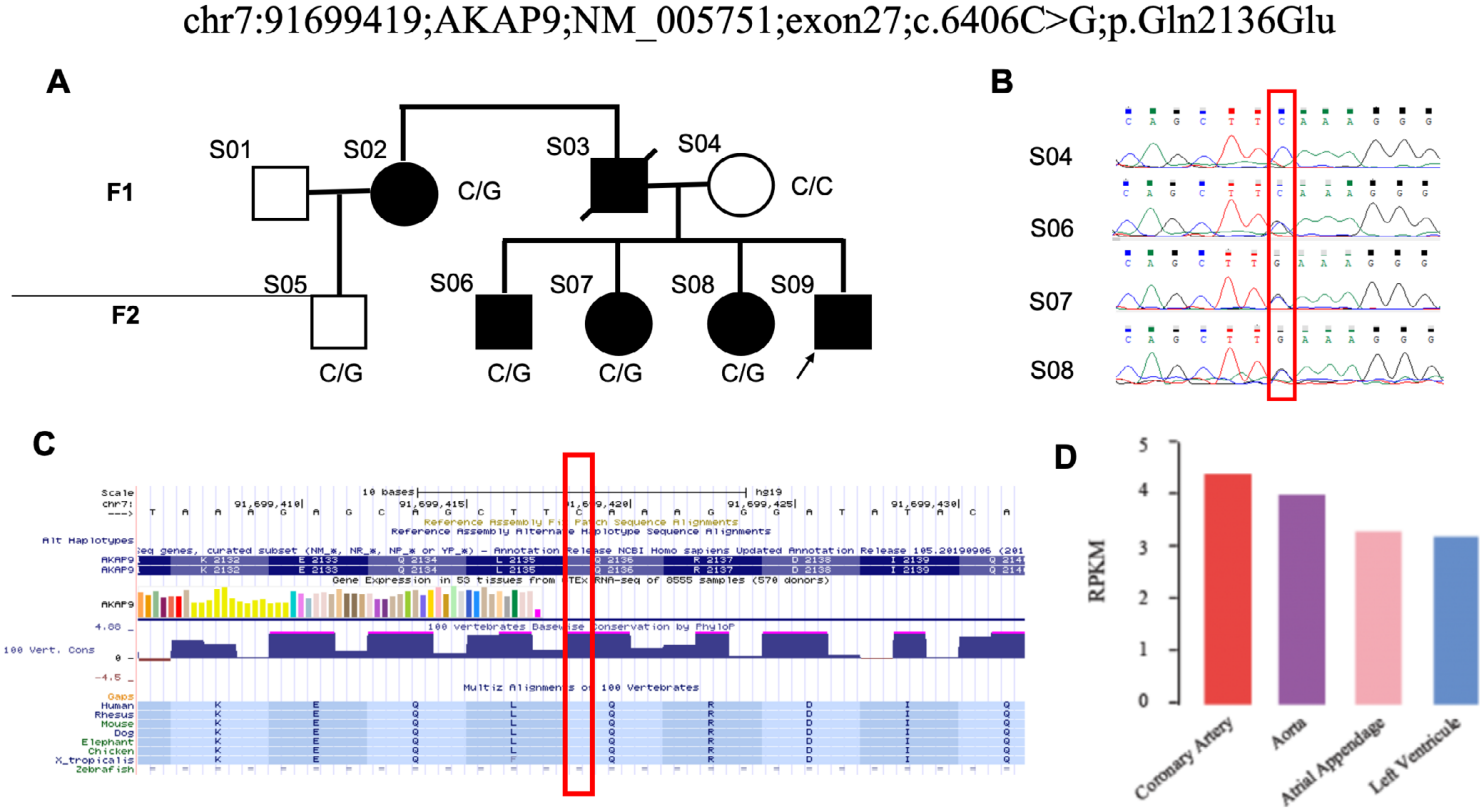
Identification of the c.6406C>G variant in *AKAP9* in a family with premature CHD. (A) Pedigree mapping of the premature CVD variant locus cosegregating with disease. This figure shows the pedigree of the proband (arrow) and her family. The solid symbols indicate the affected family members with onset of CVD before 50 years of age; the open symbols represent the unaffected family members, and a square with a solid circle indicates a male carrier. (B) Sanger sequencing validation. (C) Q2136 is highly conserved in the vertebrate genome. (D) *AKAP9* is highly expressed in the coronary artery and aorta according to the GTEX data.

Whole blood was collected using an EDTA‐K2 anticoagulant. Genomic DNA was extracted and fragmented into 180–280 bp fragments via an ultrasonic fragmentation instrument (Covaris LE220R‐plus, Covaris, USA). Whole exome sequencing (WES) was performed for 6 patients, while 4 patients underwent both WES and Sanger sequencing for validation (Figure 2A,B). For WES, the DNA fragments were end‐repaired, A‐tailed, and ligated with paired‐end adaptors. Suitable library fragments were selected for PCR amplification (T100, Bio‐Rad [Bio‐Rad Laboratories], USA). After PCR, the libraries were hybridized with biotin‐labeled probes and captured via streptavidin‐coated magnetic beads. The captured libraries underwent another round of PCR to add index tags. Products were purified via the AMPure XP system and quantified via the Agilent Bioanalyzer 2100. DNA libraries were sequenced on an Illumina platform for paired‐end 150 bp reads (NovaSeq 6000, Illumina, USA). Raw reads were filtered and mapped to the human reference genome (hg19), and duplicated reads were removed via SAMtools. Variant calling was performed via GATK4. Variants fulfilling the following criteria were subjected to subsequent analyses (Table S1): (i) missense, nonsense, frameshift, or splicing variants; (ii) absence or rare in dbSNP (http://www.ncbi.nlm.nih.gov/snp/), 1000 Genomes (http://browser.1000genomes.org/index.html), Exome Aggregation Consortium (ExAC, http://exac.broadinstitute.org/) and in‐house databases (frequency < 1%).

## 3 Case Series

### 3.1 Patient 1 (S09)

Subject 09 was a 46‐year‐old male, identified as the proband of the family. He had been diagnosed with CAD during invasive coronary angiography performed for angina, which demonstrated severe proximal stenosis of the right coronary artery (RCA). The left main (LM) artery was patent, and the left anterior descending (LAD) and left circumflex (LCX) arteries showed only mild luminal irregularities without significant stenosis. His comorbidities included a 15‐year history of hypertension (peak blood pressure 170/100 mmHg, controlled at 120–130/80–90 mmHg with medication). He had no history of diabetes mellitus, dyslipidemia, smoking, or alcohol use. He underwent four percutaneous coronary interventions (PCI) between ages 44 and 46 for recurrent angina. Echocardiography post‐intervention demonstrated regional wall motion abnormalities, reduced left ventricular ejection fraction, mild mitral and tricuspid regurgitation, and left atrial enlargement. Figure 1 shows the ECG and angiographic findings of Patient 1 (Figure 1B,C).

### 3.2 Patient 2 (S06)

Subject 06 was a 58‐year‐old male and the older brother of Subject 09 (the proband). He had experienced exertion‐related chest tightness for 8 years. In 2017, due to worsening symptoms, he was diagnosed with CAD at Peking University People's Hospital (Beijing, China), where he underwent elective percutaneous coronary intervention with implantation of three drug‐eluting stents. He was subsequently maintained on dual antiplatelet therapy. In 2019, he experienced burning chest pain without obvious cause, relieved by nitroglycerine. He was diagnosed with “acute inferior wall MI” at a local hospital. Emergency coronary angiography revealed 50% stenosis at the caudal end of the LM artery, 60% stenosis throughout the LAD artery, 90% stenosis at the D1 ostium, a small LCX, and occlusion of the original RAC stent, and two stents were placed in the RAC. One week prior, chest tightness recurred, and coronary computed tomography angiography (CTA) suggested moderate to severe stenosis in multiple vessels, leading him to visit Anzhen Hospital. He denied hypertension or other chronic medical history. Cardiac auscultation revealed a grade 2/6 systolic murmur audible in the tricuspid and mitral valve areas. Initial echocardiogram revealed post‐percutaneous coronary intervention changes, including mild mitral regurgitation, mild tricuspid valve regurgitation, and decreased left ventricular diastolic function.

### 3.3 Patient 3 (S07)

Subject 07 was a 54‐year‐old female and the older sister of Subject 09 (the proband). She had first experienced exertional chest discomfort in 2013 but was not evaluated at that time. At age 52, she presented with recurrent chest tightness that had worsened over 2 weeks and was diagnosed with coronary artery disease (CAD). CTA performed 1 week prior to presentation demonstrated light to moderate stenosis in all three coronary vessels. Subsequent invasive coronary angiography revealed a patent LM artery, 95% stenosis of the proximal LAD artery with diffuse luminal irregularities (anterior flow TIMI Grade 3) and a myocardial bridge in the mid‐LAD segment, a small LCX, and proximal RCA occlusion. She underwent coronary artery bypass grafting for further diagnosis and treatment. Post‐surgery echocardiogram showed postcoronary artery bypass graft changes, including reduced LV diastolic function. She denied any history of hypertension, diabetes, smoking, or alcohol use. At the latest follow‐up, she was asymptomatic and maintained on high‐intensity statin therapy, aspirin, and a beta‐blocker.

### 3.4 Patient 4 (S08)

Subject 08 was a 53‐year‐old female and the older sister of Subject 09 (the proband). She developed intermittent chest tightness without obvious cause at age 51, which persisted for 2 months. The episodes were not associated with chest pain, radiation, dizziness, nausea, dyspnea, or exertion. Arteriosclerosis and mild luminal stenosis were noted. She was intermittently treated with oral aspirin and atorvastatin, and Borivi was added 5 days before presentation. For further evaluation and treatment, she visited Anzhen Hospital (Beijing, China). Echocardiography revealed mild tricuspid regurgitation, mild mitral regurgitation, and reduced left ventricular diastolic function. CTA performed at a local hospital revealed arteriosclerosis and mild luminal stenosis. She was treated intermittently with oral aspirin and atorvastatin, and Borivi was added nearly 5 days prior. The echocardiogram revealed mild tricuspid regurgitation, mild mitral regurgitation, and reduced left ventricular diastolic function.

## 4 Results

### 4.1 Clinical Characteristics of Patients With Premature CAD

In this study, most family members with CAD met the criteria for premature coronary heart disease (CHD). Although two male patients (S03 and S06) were diagnosed after the age of 55, their coronary atherosclerosis had progressed to the point of myocardial infarction at the time of diagnosis. Notably, patient S06 had experienced chest pain at the age of 46 but delayed seeking medical care until 56, by which time he presented with severe coronary artery stenosis. Therefore, the actual onset of CAD in this patient should be considered to have occurred much earlier than the documented age at diagnosis. Based on these observations, we classified this family as having premature CHD. Therefore, we classify this family as one with premature CHD. None of the patients had common risk factors for CAD, such as hypertension, diabetes, smoking, or alcohol use. While their lipoprotein(a) levels were significantly higher in the positive group, we do not consider this to be a primary contributor to the early onset of the disease. Based on these findings, we hypothesize that genetic factors may play a crucial role in the development of this condition.

### 4.2 Familial Whole‐Exome Sequencing Identified c.6406C>G in *AKAP9* as a Putative Causal Variant for Premature CAD

**FIGURE 3:**
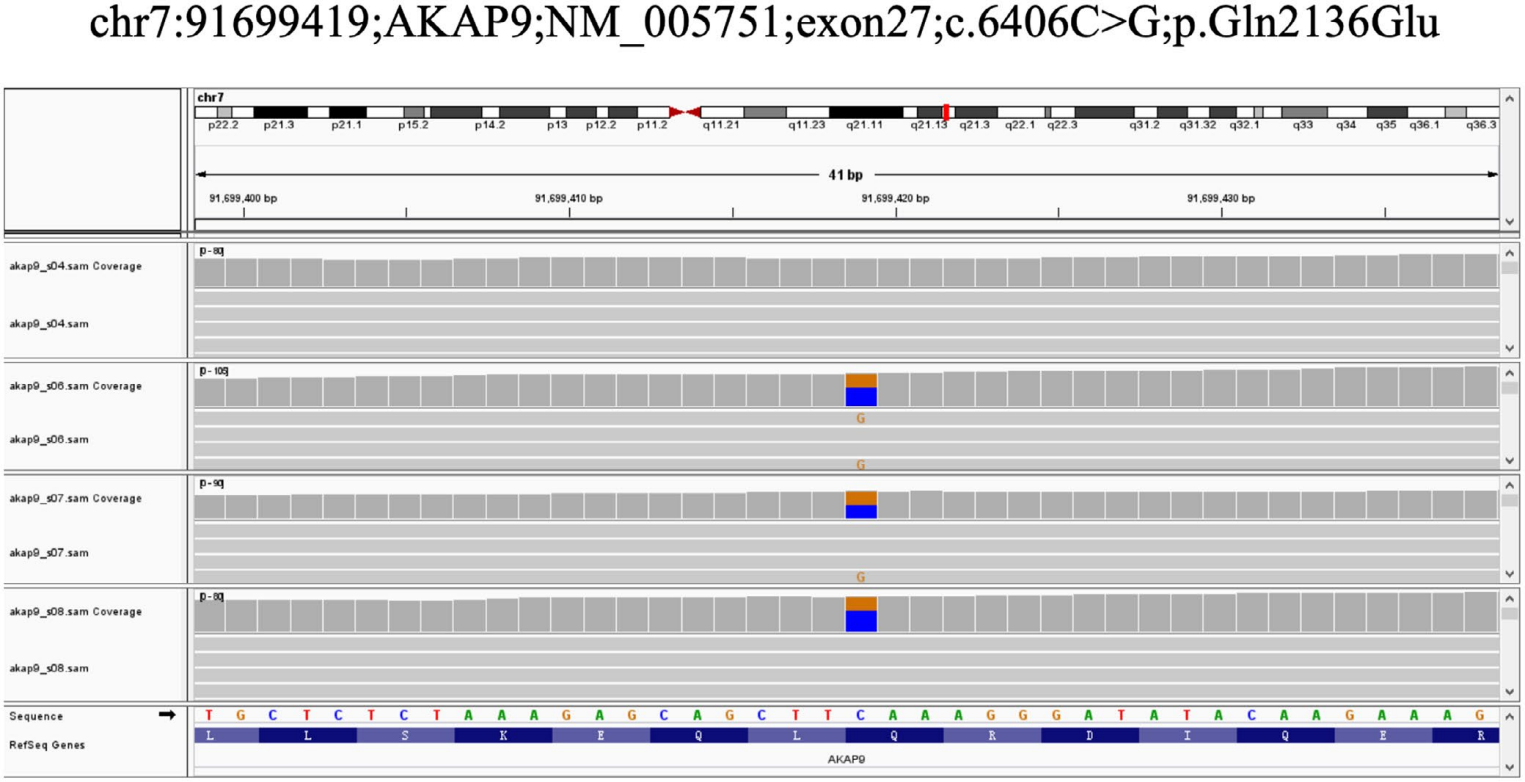
IGV of Gln2136Glu (Q2136E) in whole‐exome sequencing data of the premature CVD family.

Familial whole‐exome sequencing identified a novel c.6406C>G variant in *AKAP9*, which we propose as a potential causal variant for premature CAD. This variant was identified in three affected members (S02, S06, S07) and one unaffected member (S08), but was absent in S04 and S05. This finding was further validated by Sanger sequencing in four individuals (S04, S06, S07, S08), which confirmed the presence of the variant in S06, S07, and S08, and its absence in S04 (Figure 2A,B). In our literature review, we explored several databases, including NCBI and OMIM, and found that *AKAP9* has been linked to CVDs. Building on these findings, we hypothesized that the *AKAP9* gene carries the pathogenic variant responsible for premature CAD in this family. Our sequencing results revealed the rare c.6406C>G variant in *AKAP9*, which co‐segregated with CAD in the family. This variant was absent in public variant databases. Further validation using IGV analysis of the WES data and Sanger sequencing confirmed the presence of this variant in the family. Notably, the Q2136 position is highly conserved in the reference genome and shows high expression levels in both the aorta and heart (Figures 2 and 3).

### 4.3 Coimmunoprecipitation Indicated That p.Gln2136Glu Altered the Binding Function of AKAP9

**FIGURE 4:**
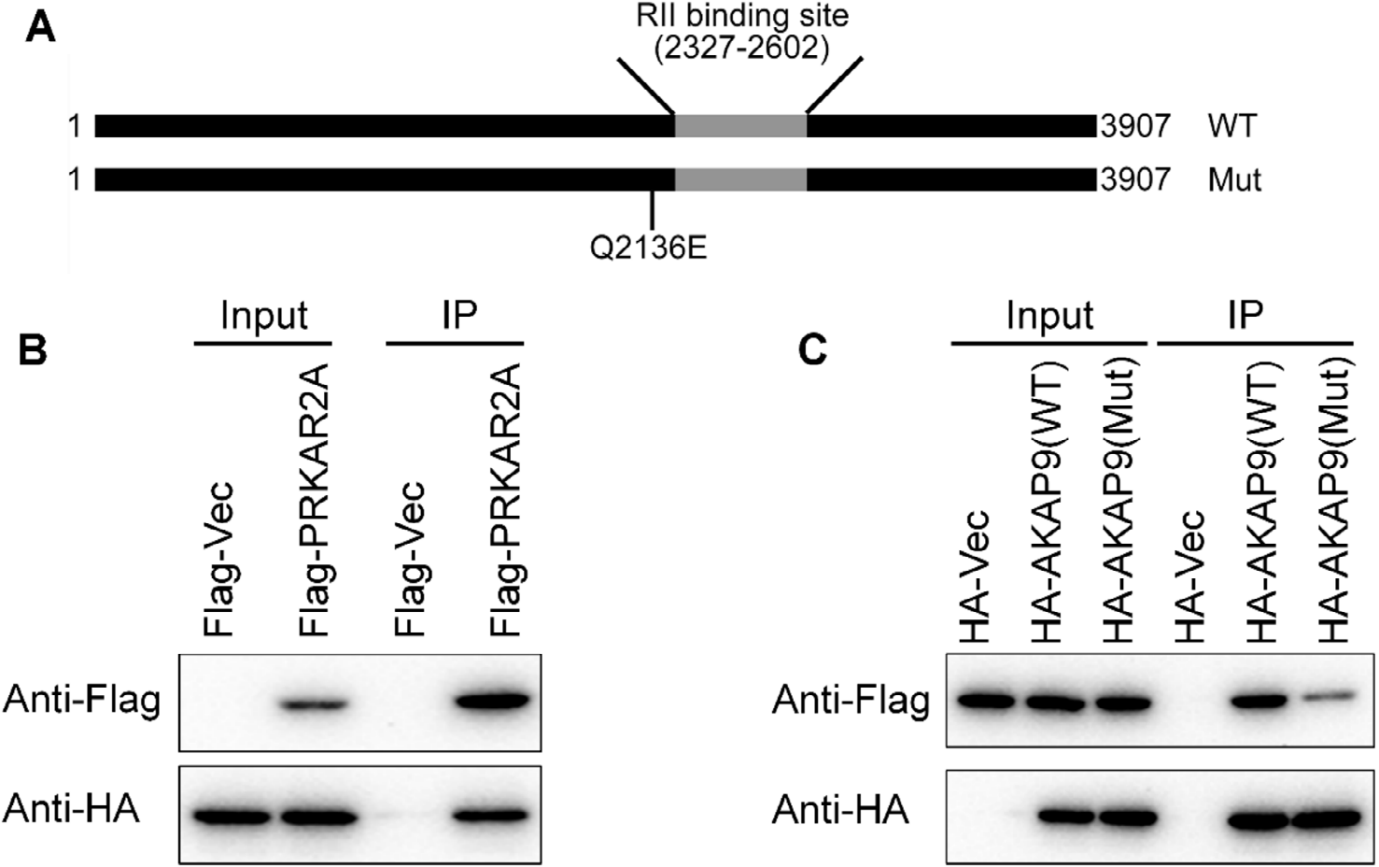
AKAP9 interacts with the PKA regulatory subunit PRKAR2A. (A) *AKAP9* structure diagram. (B) Coimmunoprecipitation experiments revealed that AKAP9 interacted with PRKAR2A. (C) Coimmunoprecipitation experiments revealed that the p.Gln2136Glu variant decreased the interaction between AKAP9 and PRKAR2A.

p.Gln2136Glu is located near the RII binding site. Coimmunoprecipitation experiments revealed that AKAP9 interacts with PRKAR2A. Furthermore, the p.Gln2136Glu variant decreased the interaction between AKAP9 and PRKAR2A (Figure 4) PRKAR2A is one of the regulatory subunits and is predominant in the heart. This subunit may interact with various A‐kinase anchoring proteins and determine the subcellular localization of cAMP‐dependent protein kinases. It has been shown to regulate protein transport from endosomes to the Golgi apparatus and subsequently to the endoplasmic reticulum (Krall et al. 1999). As a kinase responsible for MYBP‐C and TnI phosphorylation, PKA plays an important role in cardiac muscle physiology.

## 5 Discussion

A‐kinase anchoring proteins (AKAPs) are a family of more than 70 different scaffolding proteins and are key players in the spatiotemporal control of cAMP‐dependent signalling by targeting PKA and additional signaling proteins, further protein kinases and phosphatases, to specific subcellular compartments (Sherpa et al. 2024). AKAPs have no intrinsic activity of their own, but because of their crucial function, they bind protein kinase A (PKA) and other signalling proteins to engage in direct protein–protein interactions, activate the cAMP signalling pathway and target them to defined subcellular compartments (Murabito et al. 2020; Conca et al. 2025). AKAPs play essential roles in both heart and vascular physiology by coordinating complexes involved in the regulation of various processes, including endothelial‐barrier function (He et al. 2024), cardiac contraction and relaxation (Shannon et al. 2022) and action potential duration (Subramanian and Nikolaev 2023). AKAPs apparently play a role in several pathophysiological conditions in the cardiovascular system; for example, their dysregulation is associated with heart failure (Deák and Klussmann 2016). According to the HGNC database, the human AKAP (A‐kinase anchoring protein) family comprises 19 protein‐coding genes. The A‐kinase anchoring protein 9 (*AKAP9*) gene is located on chromosome 7q21.2. It contains 51 exons spanning 169.8 kb of the human genome and primarily encodes the Yotiao protein, also known as A‐kinase anchor protein 9. The PKA holoenzyme, composed of two regulatory subunits (RI or RII) and two catalytic subunits (Cα), is anchored to the KCNQ1 subunit of the IKs channel by AKAP9, thereby activating the cardiac IKs current (Zhong et al. 2024; Schwartz et al. 2021). Therefore, the *AKAP9* variant is associated with long QT syndrome (LQTS). Despite its importance in cardiac physiology and pathophysiology, there are very limited publications available showing the role of polymorphisms and/or variants in *AKAP9* in the cardiovascular system.

In this study, we identified a novel heterozygous variant in the *AKAP9* gene (c.6406C>G) in a family with premature CVD through whole‐exome sequencing (WES). Electrocardiogram (ECG) screening excluded the presence of Long QT syndrome (LQT). Bioinformatics analysis suggested that this variant is conserved and likely pathogenic. Coimmunoprecipitation experiments further demonstrated that the p.Gln2136Glu variant decreased the interaction between the AKAP9 and PRKAR2A. (Figure 4).

The *AKAP9* variant found in this study is a heterozygous variant, in line with its dominant inheritance. Although the variant gene was detected in S05 of the family, there is no definitive evidence of coronary heart disease. This may be due to his young age (37 years), as the onset age of the family members was mostly between 40 and 50 years. Therefore, we conducted a comprehensive investigation of coronary heart disease risk factors and recommended regular follow‐up of lipid profiles and CTA for this patient. The genetic mode of inheritance of CVD in this study is consistent with that described in the previously published literature (Huynh et al. 2022). Two possible mechanisms can be used to explain the presence of this variant: haploinsufficiency or the dominant‐negative effect. Haploinsufficiency is the more plausible mechanism, given that the p.Gln2136Glu variant disrupts the interaction between AKAP9 protein and PRKAR2A. The possible mechanism is that the abnormal synthesis of AKAP9 protein results in a nonfunctional truncated protein lacking the critical PKA‐RII binding domain.

### 5.1 Limitation

We acknowledge that the pedigree size is small, yielding a limited number of informative meioses; therefore, the cosegregation evidence should be considered supporting rather than definitive. Nevertheless, the observed segregation of AKAP9 c.6406C>G with the phenotype, together with functional data showing that p.Gln2136Glu reduces AKAP9–PRKAR2A interaction, suggests a relationship that is unlikely to be explained solely by lipid‐related factors. Validation in additional families and larger cohorts is warranted.

## 6 Conclusions

Our study identified the p.Gln2136Glu variant in the *AKAP9* gene as a potential genetic risk factor for premature CAD in a Chinese family. This variant may disrupt the AKAP9–PKA interaction, contributing to the pathogenesis of CAD. Our findings underscore the importance of genetic factors in familial CAD and suggest that genetic screening could enhance early diagnosis and personalized treatment strategies. Further research is needed to confirm these results and explore therapeutic approaches targeting the AKAP9–PKA pathway.

## Author Contributions

All authors contributed to the conception and design of the study. Y.J. performed genetic analyses and drafted the manuscript. M.W. and G.Q. led the conceptualization and supervision and participated in the data curation of the bioinformatic analysis. Z.X. contributed to the functional annotation of the variant and validation experiments. L.Z. assisted in patient recruitment and clinical data acquisition. Y.Y. prepared the figures, tables, and Supporting Information. C.Y. and G.S. led the conceptualization, contributed to reviewing and editing, provided overall supervision, and were responsible for acquiring funding. All authors reviewed and approved the final manuscript.

## Funding

This work was financially supported by the grant from the National Key R&D Program of China (2022YFC3600201).

## Consent

All patients and controls included in the study signed informed consent before the start of the study.

## Supporting information


**Table S1:** Candidate pathogenic variants.

## Data Availability

The data that support the findings of this study are available on request from the corresponding author C.Y. The data are not publicly available due to information that could compromise the privacy of research participants.
