# Supplementary material for: A Novel A‐Kinase‐Anchoring Protein 9 Variant in Premature Coronary Artery Disease: A Case Series
Source: Mol Genet Genomic Med. 2026 Jan 23;14(1):e70159. doi: 10.1002/mgg3.70159 (PMC12828268; doi:10.1002/mgg3.70159)
Supplement: Supplementary file 1 — Table S1: Candidate pathogenic variants. [file MGG3-14-e70159-s001.docx]

Supplementary Table1.Candidate Pathogenic Variants

| Candidate Pathogenic Variant | Related Diseases | Expression in Heart (RPKM) | Conservation | Low Frequency | Functional Impact |
| --- | --- | --- | --- | --- | --- |
| IFT70B_2q31.2_178416320_178416320_C_T_"nonsynonymous SNV" | - | - | √ | √ | SIFT: 0.01(Damaging) |
|  |  |  |  |  |  |
|  |  |  |  |  | PolyPhen2_HDIV: 1 (Damaging) |
| ITPRID2_2q31.3_182785377_182785377_T_G_"nonsynonymous SNV" | - | 1.919 | √ | √ | SIFT: 0.14(Tolerated) |
|  |  |  |  |  |  |
|  |  |  |  |  | PolyPhen2_HDIV: 1 (Damaging) |
| FSIP2_2q32.1_186668726_186668726_T_C_"nonsynonymous SNV" | Spermatogenic Failure 34 | 0.024 | √ | √ | SIFT: 0.03(Damaging); |
|  |  |  |  |  |  |
|  |  |  |  |  | PolyPhen2_HDIV: - |
| ANKAR_2q32.2_190585420_190585420_C_T_"nonsynonymous SNV" | - | 0.311 | √ | √ | SIFT: 0.01(Damaging) |
|  |  |  |  |  |  |
|  |  |  |  |  | PolyPhen2_HDIV:- |
| AKAP9_7q21.2_91699419_91699419_C_G_"nonsynonymous SNV" | LONG QT SYNDROME 11 | 0.66 | √ | √ | SIFT: 0.26(Tolerated) |
|  |  |  |  |  |  |
|  |  |  |  |  | PolyPhen2_HDIV: 0.999 (Damaging) |
| ZSCAN25_7q22.1_99220196_99220196_C_G_"nonsynonymous SNV" | - | 0.344 | √ | √ | SIFT: 0.32(Tolerated) |
|  |  |  |  |  |  |
|  |  |  |  |  | PolyPhen2_HDIV: 0.622 (Polymorphic) |
| MMS19_10q24.1_99221826_99221826_T_C_"nonsynonymous SNV" | - | 2.265 | √ | √ | SIFT: 0.75(Tolerated) |
|  |  |  |  |  |  |
|  |  |  |  |  | PolyPhen2_HDIV: 0.825 (Polymorphic) |
| ABCC2_10q24.2_101567955_101567955_T_G_"nonsynonymous SNV" | Dubin-Johnson Syndrome | 0 | √ | √ | SIFT: 0 (Damaging) |
|  |  |  |  |  |  |
|  |  |  |  |  | PolyPhen2_HDIV: 0.399 (Benign) |
| NOLC1_10q24.32_103917272_103917272_A_G_"nonsynonymous SNV" | - | 3.535 | √ | √ | SIFT: 0.06(Tolerated) |
|  |  |  |  |  |  |
|  |  |  |  |  | PolyPhen2_HDIV: 0.873 (Polymorphic) |
| PIGB_15q21.3_55619808_55619808_A_G_"nonsynonymous SNV" | Developmental And Epileptic Encephalopathy 80 | 0.459 | √ | √ | SIFT: 0.22(Tolerated) |
|  |  |  |  |  |  |
|  |  |  |  |  | PolyPhen2_HDIV: 0.319 (Benign) |
| TLNRD1_15q25.1_81294793_81294793_C_T_"nonsynonymous SNV" | - | - | √ | √ | SIFT: 0.38 (Tolerated) |
|  |  |  |  |  |  |
|  |  |  |  |  | PolyPhen2_HDIV: 0.662 (Polymorphic) |
| GSE1_16q24.1_85690931_85690931_C_T_"nonsynonymous SNV" | - | 0.063 | √ | √ | SIFT: 0.02(Damaging) |
|  |  |  |  |  |  |
|  |  |  |  |  | PolyPhen2_HDIV: 0.967 (Damaging) |
| LYPD3_19q13.31_43967413_43967413_C_T_"nonsynonymous SNV" | Atrial Septal Defect、Mitochondrial Trifunctional Protein Deficiency、Aortic Aneurysm | 0.22 | √ | √ | SIFT: 0.08(Tolerated) |
|  |  |  |  |  |  |
|  |  |  |  |  | PolyPhen2_HDIV: 1(Damaging) |
